# Early results of a real-world series with two transapical transcatheter mitral valve replacement devices

**DOI:** 10.1007/s00392-020-01757-z

**Published:** 2020-10-19

**Authors:** S. Ludwig, D. Kalbacher, N. Schofer, A. Schäfer, B. Koell, M. Seiffert, J. Schirmer, U. Schäfer, D. Westermann, H. Reichenspurner, S. Blankenberg, E. Lubos, L. Conradi

**Affiliations:** 1 Department of Cardiology, University Heart and Vascular Center Hamburg, Martinistrasse 52, 20246 Hamburg, Germany; 2 Partner Site Hamburg/Kiel/Lübeck, German Centre for Cardiovascular Research (DZHK), Hamburg, Germany; 3 Department of Cardiovascular Surgery, University Heart and Vascular Center Hamburg, Hamburg, Germany; 4 Marienkrankenhaus Hamburg, Department of Cardiology, Angiology and Intensive Care, Hamburg, Germany

**Keywords:** Transcatheter mitral valve replacement, Mitral regurgitation, Compassionate use

## Abstract

**Aims:**

Transcatheter mitral valve replacement (TMVR) with dedicated devices promises to fill the treatment gap between open-heart surgery and edge-to-edge repair for patients with severe mitral regurgitation (MR). We herein present a single-centre experience of a TMVR series with two transapical devices.

**Methods and results:**

A total of 11 patients were treated with the Tendyne™ (*N *= 7) or the Tiara™ TMVR systems (*N* = 4) from 2016 to 2020 either as compassionate-use procedures or as commercial implants. Clinical and echocardiographic data were collected at baseline, discharge and follow-up and are presented in accordance with the Mitral Valve Academic Research Consortium (MVARC) definitions.

The study cohort [age 77 years (73, 84); 27.3% male] presented with primary (*N* = 4), secondary (*N* = 5) or mixed (*N* = 2) MR etiology. Patients were symptomatic (all NYHA III/IV) and at high surgical risk [logEuroSCORE II 8.1% (4.0, 17.4)]. Rates of impaired RV function (72.7%), severe pulmonary hypertension (27.3%), moderate or severe tricuspid regurgitation (63.6%) and prior aortic valve replacement (63.6%) were high. Severe mitral annulus calcification was present in two patients. Technical success was achieved in all patients. In 90.9% (*N* = 10) MR was completely eliminated (i.e. no or trace MR). Procedural and 30-day mortality were 0.0%. At follow-up NYHA class was I/II in the majority of patients. Overall mortality after 3 and 6 months was 10.0% and 22.2%.

**Conclusions:**

TMVR was performed successfully in these selected patients with complete elimination of MR in the majority of patients. Short-term mortality was low and most patients experienced persisting functional improvement.

**Graphic abstract:**

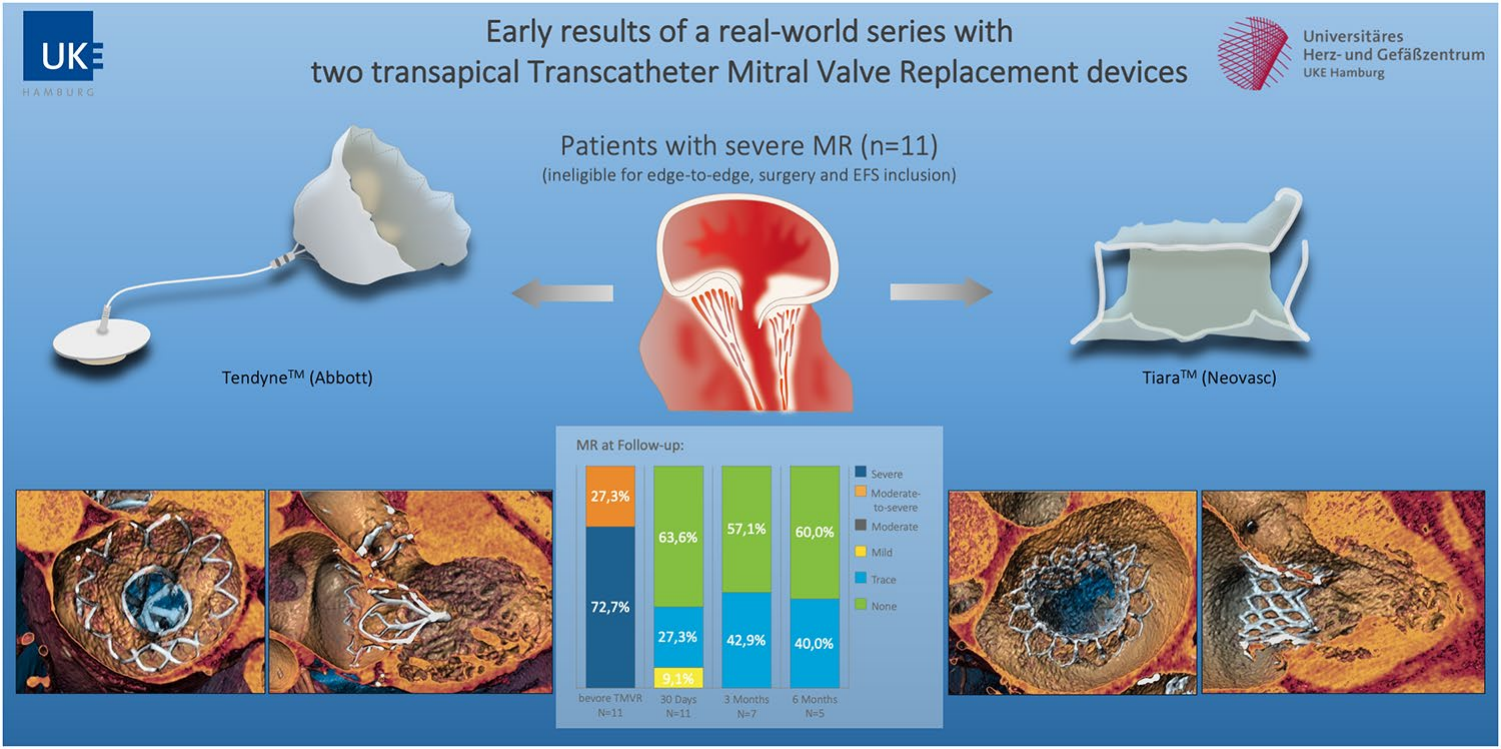

**Electronic supplementary material:**

The online version of this article (10.1007/s00392-020-01757-z) contains supplementary material, which is available to authorized users.

## Introduction

In recent years, transcatheter mitral valve replacement (TMVR) has evolved to an alternative treatment option for high-risk patients suffering from severe mitral regurgitation (MR) [1, 2]. Several ongoing early feasibility trials have shown favourable procedural and short-term outcomes with different dedicated TMVR systems in selected patient populations [3, 4]. However, a large portion of severe MR patients has so far been precluded from TMVR due to strict anatomical or clinical study exclusion criteria [5, 6]. If these patients rejected for conventional endovascular MR therapy are continuously treated medically following TMVR screening failure, notably high mortality rates after screening initiation have been reported [7–9].

While some studies suggest that TMVR is safe and feasible in patients with sensitive anatomical conditions, such as severe mitral annular calcification (MAC) or prior aortic valve replacement [10, 11], real-world data of patients treated with TMVR outside of feasibility trials are scarce. With this study, we present a single-centre experience of 11 patients with severe MR treated with two different dedicated transapical TMVR devices, either as part of a compassionate-use (CU) program or as early commercial (CE) implants.

## Methods

### Study population

From 2016 to 2020, a total of 115 patients with moderate-to-severe or severe MR were screened for TMVR at the University Heart and Vascular Center Hamburg, Hamburg, Germany. Of these, 35 patients underwent TMVR with four dedicated transapical or transseptal devices [Tendyne™ (Abbott Vascular, Abbott Park, IL, USA), Tiara™ (Neovasc Inc., New Brighton, MN, USA), CardiAQ™ (Edwards Lifesciences, Irvine, CA, USA), HighLife Valve™ (HighLife Medical, Paris, France)]. Twenty-two patients were treated as part of early feasibility trials. Ten patients received TMVR as part of CU programs (Tendyne™, Tiara™, CardiAQ™) and 3 patients were treated as CE implants (only Tendyne™). For the present study, we excluded patients included in early feasibility trials and those treated with devices no longer available (i.e. CardiAQ™). Hence, we report a series of 11 patients treated with either the Tendyne™ TMVR system (*N* = 7) or the Tiara™ TMVR system (*N* = 4). Both TMVR devices were implanted via transapical access.

### Data acquisition and follow-up

Baseline, procedural and discharge information as well as survival data were obtained from in-house information as part of clinical routine and presented in accordance with the mitral valve academic research consortium (MVARC) definitions. All patients provided written informed consent for device implantation and data acquisition. The study was conducted in accordance with the Declaration of Helsinki.

### Pre-procedural screening

All patients with severe MR were primarily screened for endovascular edge-to-edge repair. Severe leaflet calcification, small valve orifice, high transvalvular gradients and large coaptation gap represent the most frequent causes for the decision against endovascular edge-to-edge repair. The screening process for the assessment of TMVR eligibility comprised multislice computed tomography (MSCT) and echocardiography (transthoracic and transesophageal). Common reasons for anatomical TMVR ineligibility were small left ventricle (LV) dimensions, high risk of left ventricular outflow tract (LVOT) obstruction, severe circumferential MAC and annular size. A detailed description of the screening process including the decision of an interdisciplinary heart team has been given elsewhere [5]. Reasons for the decision against endovascular edge-to-edge repair for all patients and early feasibility study exclusion criteria are given in Supplementary Table 1.

### Echocardiography and MSCT

Transthoracic and transesophageal echocardiography were performed in all patients to assess MR severity and etiology. Evaluation and grading of MR were performed in line with the 2017 ESC/EACTS guidelines for the management of valvular heart disease and documented according to a standardized protocol [12]. Full cardiac cycle MSCT was performed in every patient. Dimensions of the mitral valve annulus and the LV were assessed at 30% (end-systole) and 75% (mid-to-end diastole) of the cardiac cycle with a dedicated software (3mensio Structural Heart V10.0, Pie Medical Imaging, Maastricht, Netherlands). The mitral valve annulus was measured according to a D-shaped annulus concept, as described before [13].

### Tendyne™ TMVR system

The Tendyne™ TMVR system is a self-expanding device with transapical delivery approach. It consists of a nitinol-based, dual-frame structure with an outer frame contoured to the shape of the mitral annulus and a circular inner frame holding the tri-leaflet bovine pericardial valve. The Tendyne™ device is characterized by a high-molecular-weight polyethylene tether that connects the prosthesis to the apex where it is attached to an epicardial pad. There are multiple valve size configurations ranging from AP diameter 29–41 mm and CC diameter 34–53 mm [1, 14, 15].

### Tiara™ TMVR system

The Tiara™ TMVR device is a D-shaped, nitinol alloy-based, self-expanding device consisting of a frame and a tri-leaflet bovine pericardial valve. It is implanted via transapical access. Three ventricular anchors (two anterior, one posterior) secure fixation of the valve onto the fibrous trigons and the posterior part of the annulus. A circular atrial skirt helps to seal and stabilize the device from the atrial side of the mitral annulus and the aortomitral continuity. Currently, there are two Tiara™ device sizes: 35 mm (AP 26.5–30.0 mm, CC 31.0–35.0 mm, annulus area 6.5–9.0 cm^2^) and 40 mm (AP 30.0–34.0 mm, CC 35.0–40.0 mm, annulus area 9.0–12.0 cm^2^) [1, 16, 17].

### Statistical analysis

Continuous variables were shown as median [interquartile range (IQR)] and binary variables were shown as absolute numbers and percentages. Echocardiographic parameters before and after TMVR were compared using a paired sample *t* test.

## Results

### Clinical baseline characteristics

Detailed clinical baseline characteristics of the study population (*n* = 11) are given in Table 1. The patients (77 years [73, 84], male *N* = 3) presented with high rates of impaired renal function (*N* = 10, 90.9%), immunosuppression (*N* = 5, 45.5%) and concomitant atrial fibrillation (*N *= 9, 81.8%). Prior cardiac surgery had been performed in 36.4% (*N* = 4), of whom 18.2% (*N* = 2) were treated by coronary artery bypass grafting (CABG). 63.6% (*N* = 7) of the study population had previously undergone aortic valve replacement (*N* = 5, 45.5% interventional; *N* = 2, 18.2% surgical). All patients were symptomatic according to New York Heart Association (NYHA) classes III (*N* = 10, 90.9%) or IV (*N* = 1, 9.1%). The above conditions translated into elevated surgical risk as assessed by logEuroSCORE II 8.1% (4.0, 17.4) and STS PROM 4.1% (2.6, 7.3).

**Table 1:** Clinical baseline characteristics of the study population (*n* = 11)

Clinical baseline characteristics	*n* = 11
Age (years)	77 (73, 84)
Female gender	8 (72.7)
Body mass index (kg/m^2^)	25.5 (21.6, 29.5)
logEuroSCORE II (%)	8.1 (4.0, 17.4)
STS PROM (%)	4.1 (2.6, 7.3)
Diabetes	1 (9.1)
COPD	2 (18.2)
Impaired renal function (GFR < 50 ml/min)	10 (90.9)
Chronic haemodialysis	2 (18.2)
Immunosuppression	5 (45.5)
Atrial fibrillation	9 (81.8)
Prior myocardial infarction	2 (18.2)
Prior cardiac surgery	4 (36.4)
Prior CABG	2 (18.2)
Prior aortic valve replacement (TAVI or SAVR)	7 (63.6)
Prior TAVI	5 (45.5)
Prior SAVR	2 (18.2)
Heart failure hospitalization (last year)	10 (90.9)
NYHA stage III	10 (90.9)
NYHA stage IV	1 (9.1)

*CABG* coronary artery bypass graft, *COPD* chronic obstructive pulmonary disease, *GFR* glomerular filtration rate, *NYHA* New York Heart Association, *SAVR* surgical aortic valve replacement, *STS PROM* Society of Thoracic Surgeons Predicted Risk of Mortality, *TAVI* transcatheter aortic valve replacement

### Echocardiographic and MSCT parameters

Echocardiographic and MSCT parameters are presented in detail in Table 2. Etiology of MR was functional (FMR), degenerative (DMR) or mixed FMR/DMR in 45.5% (*N* = 5), 36.4% (*N* = 4) and 18.2% (*N* = 2), respectively. Severity of MR was moderate-to-severe (3 +) in 27.3% (*N* = 3) and severe (4 +) in 72.7% (*N* = 4) of the patients. Left ventricular ejection fraction (LVEF) was 45.0% (35.0, 53.5), while in 18.2% (*N* = 2) of the patients LVEF was severely reduced. Reduced stroke volume index (SVI < 35 ml/m^2^) at baseline was found in more than half of the study population (*N* = 6, 54.5%). Impaired right ventricular (RV) function was present in 72.7% (*N* = 8) of all patients with a high median systolic pulmonary artery pressure (sPAP) of 48.0 mmHg (36.0, 55.0). Moderate or severe tricuspid regurgitation (TR) was assessed in 63.6% (*N* = 7) of the study population.

**Table 2:** Echocardiographic and computed tomography parameters

Echocardiographic parameters	*n* = 11
MR 3 + (%)	3 (27.3)
MR 4 + (%)	8 (72.7)
FMR (%)	5 (45.5)
DMR (%)	4 (36.4)
Mixed FMR/DMR (%)	2 (18.2)
Pmean (mmHg)	4.0 (3.0, 4.8)
EROA (cm^2^)	0.39 (0.31, 0.52)
LVEDV (mL)	114.6 (92.1, 163.8)
LVEF (%)	45.0 (35.0, 53.5)
Severely reduced LVEF ≤ 30%	2 (18.2)
SVI (ml/m^2^)	31.2 (21.2, 38.6)
Reduced SVI < 35 ml/m^2^	6 (54.5)
TAPSE (mm)	16.0 (14.5, 17.0)
Impaired RV function (TAPSE ≤ 17 mm)	8 (72.7)
Systolic PAP (mmHg)	48.0 (36.0, 55.0)
Systolic PAP ≥ 55 mmHg	3 (27.3)
≥ Moderate TR	7 (63.6)

Computed tomography parameters	
CC diameter (mm)	38.9 (33.7, 42.8)
AP diameter (mm)	34.7 (31.7, 36.3)
Dmean^a^ (mm)	38.1 (32.4, 39.4)
Mitral annulus perimeter (mm)	127.0 (118.5, 131.9)
Mitral annulus area (cm^2^)	12.1 (10.5, 12.9)
CTA length (mm)	91.7 (82.5, 99.1)
Aorto-mitral angulation (°)	134.3 (118.5, 138.5)
Any MAC	8 (72.7)
Severe circumferential MAC	2 (18.2)

Computed tomography parameters are presented as end-systolic measurements

*AP* anterior–posterior, *CC* intercommissural, *CTA* center-to-apex, *Dmean* mean mitral annulus diameter, *DMR* degenerative mitral regurgitation, *EROA* effective regurgitant orifice area, *FMR* functional mitral regurgitation, *LVEDD* left ventricular end-diastolic diameter, *LVEDV* left ventricular end-diastolic volume, *LVEF* left ventricular ejection fraction, *MAC* mitral annulus calcification, *MR* mitral regurgitation, *PAP* pulmonary artery pressure, *Pmean* mean transvalvular gradient, *RV* right ventricular, *SVI* stroke volume index, *TAPSE* tricuspid annular plane systolic excursion, *TR* tricuspid regurgitation

^a^Dmean = (IC diameter + AP diameter) / 2

Mitral annulus intercommissural (CC) diameters of all treated patients, as assessed by pre-procedural MSCT, ranged from 30.0 mm to 47.6 mm (measured in end-systole) with a median of 38.9 mm (33.7, 42.8). Aorto-mitral angulation was 134° (119, 139). Any MAC was present in 72.7% (*N* = 8) of all patients. In two patients, severe circumferential MAC was identified.

### Procedural and in-hospital outcome

Procedural parameters are given in Supplementary Table 2. Seven patients were treated with the Tendyne™ valve and four patients underwent Tiara™ implantation. All procedures (*N* = 11) were conducted via transapical access with transesophageal echocardiography guidance under general anesthesia. Technical success was achieved in all procedures (100.0%) and all patients were extubated immediately after the procedure. Relevant LVOT obstruction was detected neither echocardiographically nor invasively. No patient required mechanical circulatory support during the procedure and there were no cases of conversion to open-heart surgery. Invasively measured cardiac index, assessed by Swan Ganz catheterization with thermodilution, increased from 1.71 L/min/m^2^ (1.55, 1.98) before TMVR to 1.85 L/min (1.71, 2.38) (*p* = 0.06). There was no procedural or in-hospital mortality (0.0%). After the procedure, all patients were prescribed lifelong oral anticoagulation with a vitamin K antagonist (INR 2.5–3.5) (with or without single anti-platelet therapy).

### Echocardiographic outcomes at discharge

**Fig. 1:**
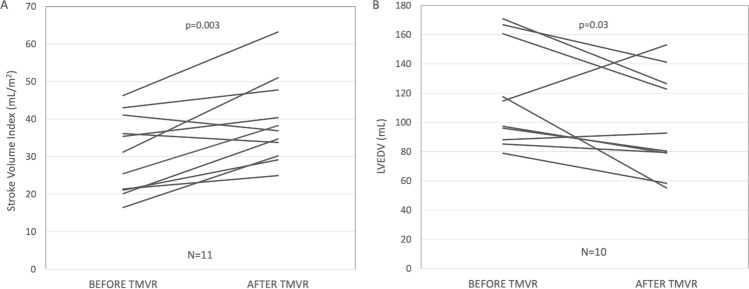
Echocardiographic changes before and after TMVR. **a** Stroke volume index (SVI) (mL/m^2^). **b** Left ventricular end-diastolic volume (LVEDV). *LVEDV* left ventricular end-diastolic volume, *SVI* stroke volume index, *TMVR* transcatheter mitral valve replacement

Detailed echocardiographic findings at discharge are given in Table 3. Mean transprosthetic gradients were low with a median of 3.0 mmHg (3.0, 5.0). One patient (9.1%) was diagnosed with mild paravalvular leakage (PVL), three patients (27.3%) had trace valvular MR and in 7 patients (63.6%) no residual MR was detected. SVI after TMVR increased from 31.2 mL/m^2^ (21.2, 38.6) to 36.9 mL/m^2^ (32.0, 44.1) (*p* = 0.003) (Fig. 1a). Moreover, echocardiography after TMVR revealed a significant reduction in left ventricular end-diastolic volumes (LVEDV) (median 114.6 mL to 86.5 mL, *p* = 0.03) (Fig. 1b) and sPAP (median 48 mmHg to 38 mmHg, *p* = 0.008). No significant post-procedural changes were found for LVEF or RV function.

**Table 3:** MVARC 30-day and echocardiographic outcomes at discharge

Echocardiographic outcomes	
Pmean (mmHg)	3.0 (3.0, 5.0)
> trace PVL	1 (9.1)
Mean LVOT gradient (mmHg)	1.8 (1.7, 3.6)
Peak LVOT gradient (mmHg)	3.6 (3.3, 6.4)
LVEDV (mL)	86.5 (79.1, 125.5)
LVEF (%)	39.0 (35.0, 50.0)
SVI (mL/m^2^)	36.9 (32.0, 44.1)
TAPSE	15 (12.3, 16.0)
Systolic PAP (mmHg)	38.0 (35.0, 44.0)
≥ Moderate TR	5 (45.5)

MVARC 30-day outcomes	*n* = 11
30-Day mortality	0 (0.0)
Valve embolization/migration	0 (0.0)
Valve thrombosis	0 (0.0)
MV surgery/reintervention	0 (0.0)
Myocardial infarction	0 (0.0)
Disabling stroke	0 (0.0)
Access-site complications	2 (18.2)
Low cardiac output/inotrope therapy	1 (9.1)
Major/life-threatening bleeding	2 (18.2)
Renal failure ( ≥ AKIN 2)	1 (9.1)
New-onset atrial fibrillation	1 (9.1)

*AKIN* Acute Kidney Injury Network, *HF* heart failure, *LVEDV* left ventricular end-diastolic volume, *LVEF* left ventricular ejection fraction, *LVOT* left ventricular outflow tract, *MV* mitral valve, *PAP* pulmonary artery pressure, *Pmean* mean transvalvular gradient, *PVL* paravalvular leakage, *SVI* stroke volume index, *TAPSE* tricuspid annular plane systolic excursion, *TR* tricuspid regurgitation

### MVARC 30-day outcomes

Detailed MVARC 30 day outcomes 30 days after TMVR are also displayed in Table 3. After 30 days all-cause mortality was still 0.0%. Severe complications such as valve embolization or migration, valve thrombosis, mitral valve reintervention, myocardial infarction or disabling stroke did not occur within 30 days after TMVR. One patient required intermittent inotrope therapy due to temporary post-procedural low cardiac output. There were two cases of MVARC major bleeding due to access-site complications: one patient developed haemothorax with need for intermittent thoracic drainage installation. Another patient experienced secondary haemorrhage from the femoral puncture site. Acute kidney failure ≥ AKIN 2 occurred in one patient requiring post-procedural haemodialysis.

### Late echocardiographic and clinical outcomes after TMVR

**Fig. 2:**
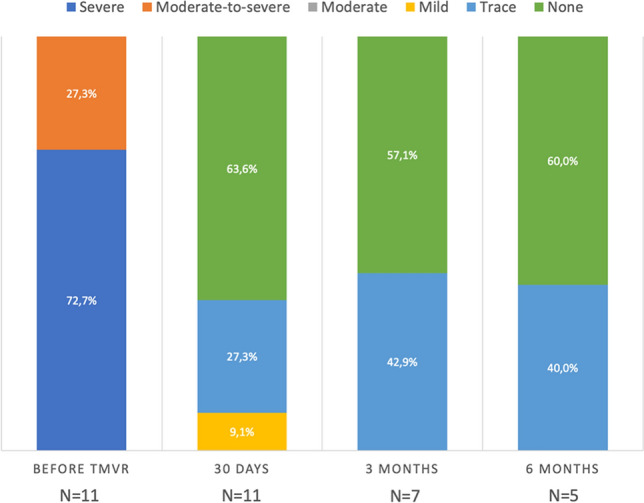
Mitral regurgitation before TMVR and at follow-up. *TMVR* transcatheter mitral valve replacement

**Fig. 3:**
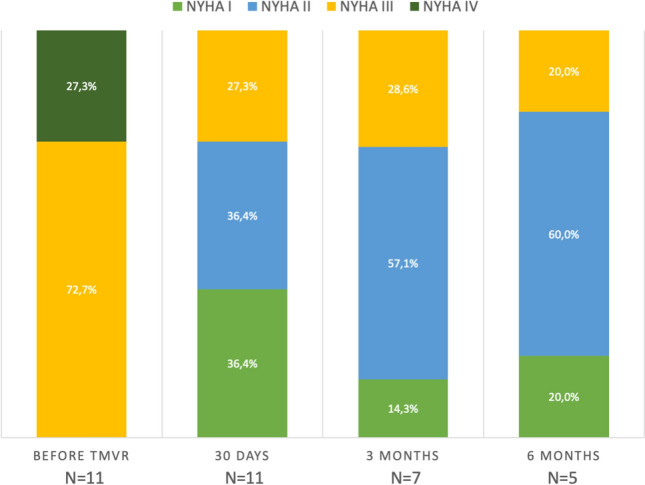
NYHA class distribution before TMVR and at follow-up. *NYHA* New York heart association, *TMVR* transcatheter mitral valve replacement

The severity of MR before TMVR and 30 days, 3 and 6 months after TMVR is demonstrated in Fig. 2. There were no cases of more than trace valvular MR. Figure 3 depicts the distribution of NYHA classes before TMVR and throughout all follow-up visits indicating functional improvement with a reduction to NYHA classes I or II in the majority of patients. After 79 and 97 days, two patients died after refractory resuscitation due to conduction disturbances in the first and due to an unknown cause in the second case resulting in all-cause mortality rates of 10.0% after 3 months and 22.2% after 6 months. All-cause mortality rate after 1 year was 33.3%, as another patient expired after diagnosis of valve thrombosis following thrombolysis-related cerebral haemorrhage after 281 days. Mortality rates 30 days, 3 months, 6 months and 1 year after TMVR are summarized in Table 4. Longer term follow-up data of more than 2 years can be reported for 3 patients (follow-up: 3.7, 2.8 and 2.3 years) treated in 2016 and 2017, all presenting with no or trace MR, mean transvalvular gradient 3–4 mmHg and NYHA stage II or III.

**Table 4:** Mortality rates 30 days, 3 months, 6 months and 1 year after TMVR

Follow-up	Mortality rates
30 days (*N* = 11)	0.0%
3 months (*N* = 10)	10.0%
6 months (*N* = 9)	22.2%
1 year (*N* = 9)	33.3%

## Discussion

**Fig. 4:**
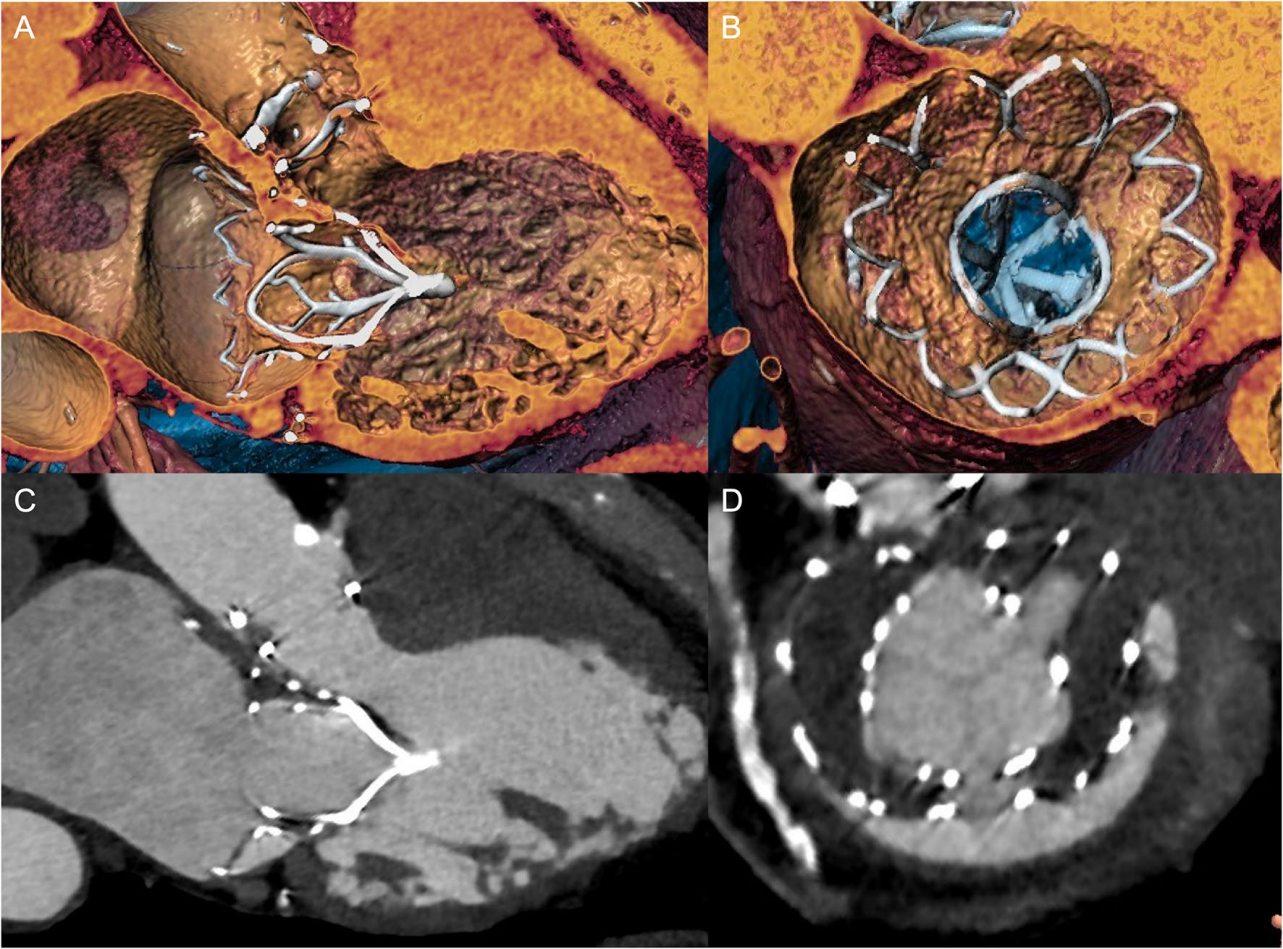
Tendyne™ (Abbott Vascular) and prior AVR. (3Mensio Structural Heart, V10.0, Pie Medical Imaging, Maastricht, Netherlands). **a** 3D reconstruction of three-chamber view. **b** 3D en face view (“surgeon’s view”) of the device. **c** 2D three-chamber view. **d ** 2D en face view of the device

**Fig. 5:**
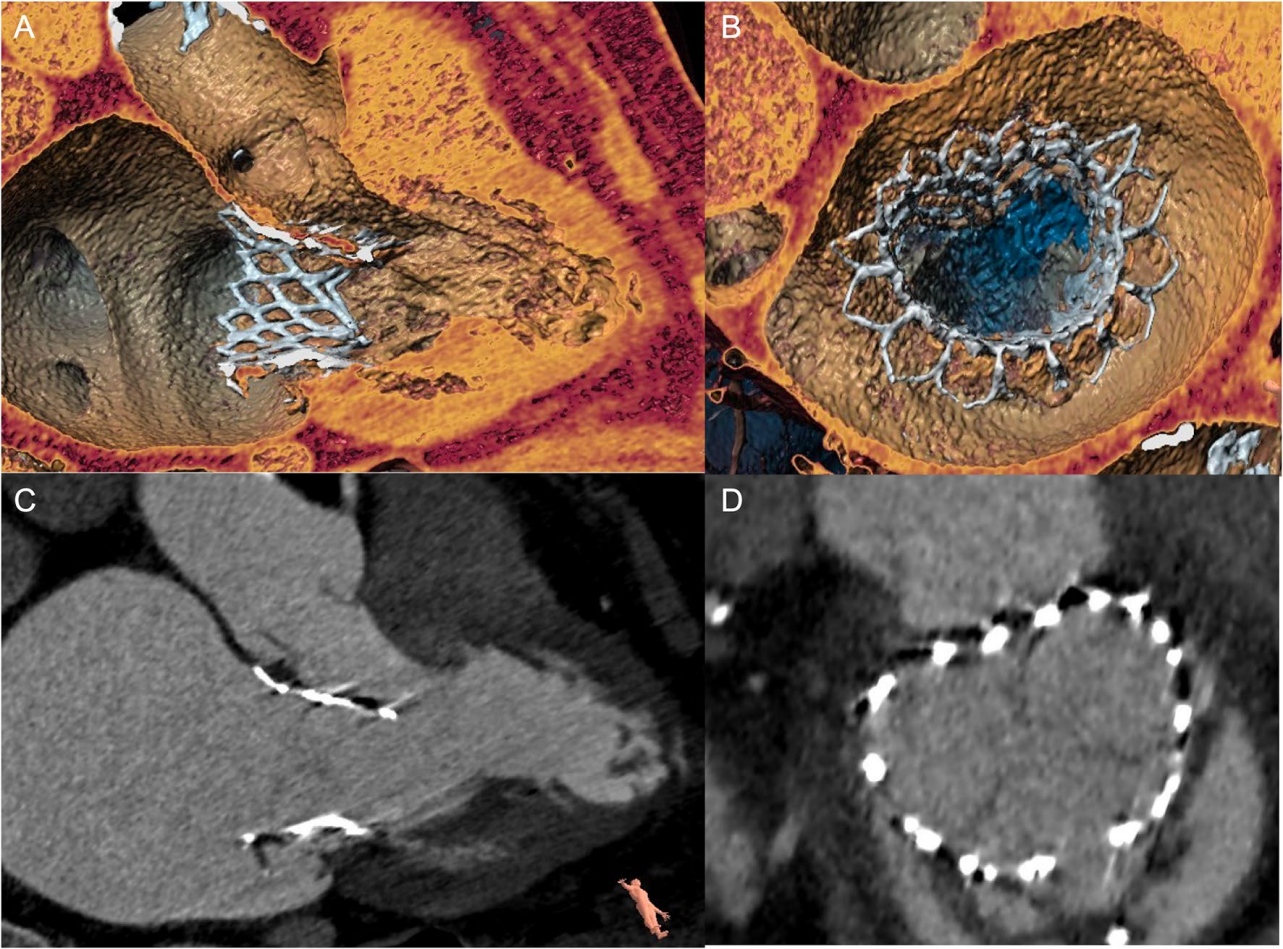
Tiara™ (Neovasc Inc.). (3Mensio Structural Heart, V10.0, Pie Medical Imaging, Maastricht, Netherlands). **a** 3D reconstruction of three-chamber view. **b** 3D en face view (“surgeon’s view”) of the device. **c** 2D three-chamber view. **d** 2D en face view of the device

This study reports one of the first series of TMVR implanted outside trial protocol-related restrictions. Patients were treated with the Tendyne™ valve (Fig. 4) or the Tiara™ valve (Fig. 5) either under a CU program or as CE implants, including the world’s first CE implants with the Tendyne™ TMVR system. Our results suggest feasibility of TMVR in patients that were excluded from ongoing TMVR trials with a low procedural event rate for implanted TMVR devices, functional improvement according to NYHA class and elimination of MR in the majority of patients.

Recently published results of early feasibility trials with dedicated TMVR devices have impressively demonstrated the potential of TMVR as a true alternative for patients ineligible for established mitral valve therapies. Data from the early feasibility study of the Tendyne mitral valve system demonstrated safety of device implantation with the Tendyne™ valve as well as functional improvement and effective reduction of MR [4]. The authors reported technical success in 96%, 30-day and 1-year mortality rates of 6.0% and 26.0%, respectively. In survivors, 88.5% experienced persisting symptom relief after 1 year. According to echocardiography at follow-up, LVEDV and sPAP decreased 1 year after TMVR [4]. Recently, the 2-year results were presented by Muller et al. with an all-cause mortality rate of 39.0% and no or trivial MR in 93.2% at 2 years after TMVR with the Tendyne™ valve [18].

The latest results of the early feasibility study of the Neovasc Tiara™ mitral transcatheter heart valve system (TIARA-I) and the Tiara™ Transcatheter Mitral Valve Replacement Study (TIARA-II) have recently been reported with high technical success, 0.0% procedural mortality and device-related mortality of 7.3% after 30 days [19]. Long-term results of these studies have not been published so far. However, resolution of MR was also observed in the majority of patients treated with the Tiara™ valve suggesting comparable findings [19].

Extensive exclusion criteria for ongoing early feasibility TMVR trials have led to high screening failure rates due to anatomical or clinical reasons [5–7, 20]. While anatomical restrictions, such as risk of LVOT obstruction, annular dimensions and ventricular restraints largely depend on valve size and ventricular profile and, therefore, may at least partly be attenuated, clinical reasons for TMVR ineligibility mostly represent cautiousness of device manufacturers and reservations to perform TMVR in clinical conditions associated with adverse outcome [21–23]. Niikura et al. describe excessive frailty, severe TR and prior aortic valve replacement as common clinical reasons for TMVR exclusion [7]. Severe pulmonary hypertension, impaired LVEF and severe MAC represent further frequent factors associated with patient denial for TMVR [5, 6, 24].

The results of the herein presented real-world experience of patients with severe MR treated with two dedicated TMVR devices outside of company-funded feasibility trials demonstrate favourable echocardiographic and functional outcomes with both implanted devices. The study population is characterized by high prevalence of pulmonary hypertension, severe TR and impaired RV function, chronic kidney injury and high rates of prior surgical or interventional aortic valve replacement. Moreover, this cohort includes patients with severe circumferential MAC. Representing major study exclusion criteria these factors would have precluded those patients from interventional MR therapy via TMVR resulting in medical therapy only, which has previously shown to yield high mortality rates after TMVR screening failure [7, 8]. Similar to the above-discussed trials with the Tendyne™ and Tiara™ valves, etiology of MR in the present study cohort was FMR [18, 19]. Our study demonstrates feasibility of TMVR in patients with the above conditions, with technical success in 100.0% and 30-day mortality of 0.0%. After TMVR, echocardiographic assessment showed an increase in SVI and decreasing LV filling volumes correlating with effective symptom relief (NYHA class I/II) in almost all patients. Moreover, we can report complete resolution of MR in the majority of patients treated, persisting after 3 and 6 months. However, 1-year mortality rate was comparatively high, which may be explained by an overall reduced life expectancy due to coexisting comorbidities.

In accordance with our results, some studies have already demonstrated feasibility of TMVR in patients with special preconditions, such as MAC and prior aortic valve replacement [10, 11]. Although these results are promising, results of ongoing trials like the MAC arm of the SUMMIT trial with the Tendyne™ valve (NCT03433274) or the results of the TIARA-I and -II (NCT02276547, NCT03039855) studies, will shed more light on this issue. Compared to other established interventional therapies targeting MR, the most important benefit achieved with TMVR may be the resolution of MR in almost all cases. Therefore, investigations addressing long-term effects of TMVR on LV remodelling and survival of patients are highly warranted. Recent studies have shown that residual MR and high mitral valve pressure gradients, especially in DMR patients, are associated with adverse outcome after endovascular edge-to-edge repair [25–27]. Prospectively, TMVR might constitute an alternative treatment option especially for patients with combined mitral valve disease (i.e. with small orifice, calcified leaflets, etc.), in whom MR elimination by edge-to-edge repair without high transvalvular gradients is unlikely.

### Limitations

The presented study is limited by its study design and sample size. First, any drawn conclusions can only serve to generate hypotheses due to the retrospective study design. Second, the presented sample size is comparatively small. However, as TMVR represents a novel therapy, this single-centre study represents the first report of patients treated successfully with TMVR outside of early feasibility trials.

## Conclusion

In this real-world series of TMVR with two dedicated devices outside trial protocols, TMVR was performed successfully in all patients and complete elimination of MR was achieved in the majority of patients. Despite a high-risk profile, short-term mortality was low and patients experienced persisting functional improvement. These results suggest that TMVR has the potential to become an alternative treatment option for a broader subset of patients.

## Electronic supplementary material

Below is the link to the electronic supplementary material.

Supplementary file1 (DOCX 16 kb)

Supplementary file2 (MP4 5443 kb)

Supplementary file3 (MP4 20417 kb)

Supplementary file4 (MP4 6475 kb)

Supplementary file5 (MP4 10681 kb)
