# Supplementary material for: Early results of a real-world series with two transapical transcatheter mitral valve replacement devices
Source: Clin Res Cardiol. 2020 Oct 19;110(3):411–20. doi: 10.1007/s00392-020-01757-z (PMC7907022; doi:10.1007/s00392-020-01757-z)
Supplement: Supplementary file 1 — Supplementary file1 (DOCX 16 kb) [file 392_2020_1757_MOESM1_ESM.docx]

**Supplementary table 1**: Decision against percutaneous MV repair and EFS exclusion criteria

| **Patient** | **Decision against percutaneous MV repair** | **EFS exclusion (Tendyne^TM^)** | **EFS exclusion (Tiara^TM^)** |
| --- | --- | --- | --- |
| Tendyne #1 | severe PML flail, large coaptation gap, calcified PML | severe TR, RV dysfunction, prior TAVI |  |
| Tendyne #2 | severely calcified PML, small orifice | RV dysfunction, prior TAVI |  |
| Tendyne #3 | small orifice, MAC, high MVPG | severe MAC |  |
| Tendyne #4 | combined MR/MS, high MVPG, PML cleft | prior TAVI |  |
| Tendyne #5 | calcified PML | RV dysfunction, prior SAVR |  |
| Tendyne #6 | extremely restrictive and short PML, AML flail | severe TR, RV dysfunction, severe PHT, prior TAVI |  |
| Tendyne #7 | failed E2E attempt due to moderate stenosis and residual MR, calcified leaflets | severe MAC, prior ViV-TAVI |  |
| Tiara #1 | short, restrictive PML |  | severe TR, CKD (eGFR<35ml/min) |
| Tiara #2 | multiple jets, elevated gradient, small orifice |  | frailty, breast cancer |
| Tiara #3 | short, very calcified PML, severe PML/AML restriction |  | high risk (STS PROM 13.5%), chronic haemodialysis, COPD, immunosuppression |
| Tiara #4 | severely calcified/immobile PML, small orifice |  | severe PHT, prior SAVR, CKD (eGFR<35ml/min) |

Abbreviations:

AML=anterior mitral leaflet, CKD=chronic kidney disease, COPD=chronic obstructive pulmonary disease, E2E=edge-to-edge, eGFR=estimated glomerular filtration rate, MAC=mitral annulus calcification, MR=mitral regurgitation, MS=mitral stenosis, MV=mitral valve, MVPG=mitral valve pressure gradient, PHT=pulmonary hypertension, PML=posterior mitral leaflet, RV=right ventricular, SAVR=surgical aortic valve replacement, STS PROM=Society of Thoracic Surgeons Predicted Risk of Mortality, TAVI=transcatheter aortic valve implantation, TR=tricuspid regurgitation, ViV=Valve-in-Valve

**Supplementary table 2**: Procedural parameters

| **Procedural parameters** | **n=11** |
| --- | --- |
| Transapical access | 11 (100.0) |
| Procedure time (min) | 150 (125, 170) |
| Technical success | 11 (100.0) |
| LVOT obstruction | 0 (0.0) |
| Mechanical circulatory support | 0 (0.0) |
| Procedural mortality | 0 (0.0) |

Abbreviations: LVOT=left ventricular outflow tract
